# Marginal bone loss in the second molar related to impacted mandibular third molars: comparison between panoramic images and cone beam computed tomography

**DOI:** 10.4317/medoral.23443

**Published:** 2020-04-09

**Authors:** Maristela Junqueira Maciel Dias, Ademir Franco, José Luiz Cintra Junqueira, Flávio Tendolo Fayad, Paulo Henrique Pereira, Anne Caroline Oenning

**Affiliations:** 1 Division of Oral Radiology, Faculdade São Leopoldo Mandic, Instituto de Pesquisas São Leopoldo Mandic, SP, Brazil

## Abstract

**Background:**

Deciding whether or not to extract third molars remains a controversial situation in dental practice. Image exams support this decision by enabling a close view of the third molar, its adjacent bone and its relationship with the second molar. This study aimed to assess and compare second molar bone loss adjacent to impacted mandibular third molar in panoramic radiographs (PAN) and cone beam computed tomography (CBCT) scans.

**Material and Methods:**

A sample of 70 patients was selected (n=124 teeth). Each patient had a set of a panoramic radiograph and CBCT scans consecutively taken for dental treatment purposes. In PAN and CBCT, mandibular third molars were classified based on their position and bone loss of the adjacent second molar. Agreement between PAN and CBCT scans was assessed and quantified.

**Results:**

Outcomes of bone loss assessment were different between PAN and CBCT scans (*p*<0.05). Bone loss was found in 62.9% of the PAN, while in CBCT scans it was found in 80%. In particular, nearly 29% (n=27) of the teeth that were classified without bone loss in PAN were classified with bone loss in CBCT scans. Mesioangular and horizontal third molars had a statistically significant association with bone loss of the adjacent second molars (*p*<0.05). In general, PAN underestimated the severity of bone loss compared to CBCT scans (*p*<0.05).

**Conclusions:**

Diagnosing second molar bone loss due to impaction of adjacent third molar in PAN may be challenging because of false negatives. Impacted third molars justify preoperative CBCT scans if second molar bone loss needs to be precisely assessed for a more detailed and reliable treatment plan.

** Key words:**Bone, CBCT, panoramic radiograph, third molar.

## Introduction

Periodontal health and prognosis of teeth directly depend on the available bone support (1). Bone loss may be induced by several factors, such as smoking habit (2), level of education (2) and the presence of adjacent impacted tooth (3). The latter plays an important part in the contemporary dental practice because tooth impaction is often found, especially involving mandibular third molars (4). Recent statistics calculated from a systematic review and meta-analysis of over 30 studies revealed a mean prevalence of 24.40% of third molar impaction worldwide (5). Among the negative effects of third molars in periodontal health is the increase of periodontal pockets distal to second molars (6). Scientific evidences showed that third molars represent a higher risk to second molar pathology and loss when impacted on soft tissue (7).

Assessing the relationship between second and third molars is essential before taking decisions in practice. Panoramic radiographs and cone beam computed tomography (CBCT) scans enable bi- and three-dimensional assessments of the dentomaxillofacial structures, respectively (8). In theory, more information may be retrieved from the detailed multiplanar navigation feasible in CBCT, which can offer support for determining specific decisions (9). However, panoramic radiographs involve less ionizing radiation for image acquisition and its diagnostic accuracy must be tested in face of CBCT’s performance. Using panoramic radiographs for investigating bone loss in the interface of second and third molars may be challenging because of the inherent image distortion and overprojection of structures – usually the crown of the impacted third molar and the distal surface of the second molar.

Based on the exposed justification, this study aimed at assessing and comparing the detection and severity of second molar bone loss adjacent to impacted third molars between panoramic radiographs and CBCT scans.

## Material and Methods

The study was structured and presented according to the guidelines of the Strengthening the Reporting of Observational Studies in Epidemiology (STROBE) (10).

- Participants and settings

The sample consisted of panoramic radiographs and CBCT scans taken from the same patients within a maximum time interval of 30 days. CBCT scanning after panoramic radiograph was justified for therapeutic purposes – all the patients were scheduled for third molar removal and had the position of their third molars and the inherent anatomic relation with adjacent teeth and mandibular canal three-dimensionally analyzed via CBCT scans. The eligibility criteria for sampling included male and female patients, aged between 18 and 60 years, with at least one impacted third molar. Patients with cysts or tumor lesions were excluded, as well as patients with missing second molars adjacent to the impacted third molars. In order to support the sampling eligibility criteria with scientific rationale, sample size calculation was performed. According to the test, sixty patients would result in sample power of 0.8 for effects above 0.33 (considering statistical significance of 0.05). Hence, sampling was conducted and resulted in 70 patients (n: 124 third molars) aged between 18 and 57 years (mean age 25.7 years ± 8.6). Thirty-seven patients were females (52.9%) and 33 were males (47.1%).

- Data source

Image acquisition for panoramic radiographs was performed with OP200 device (Instrumentarium Dental™, Tuusula, Finland) set with 66 kVp, 8 mA and 14 seconds of exposure. For CBCT scanning, iCAT unit (Imaging Sciences International, Hatfield, PA, USA) was used. CBCT setting included field of view of 16 x 13cm, voxel size of 0.25 mm, 120 kVp and 36 mA. Image analysis was performed in consensus by two experienced Maxillofacial Radiologists, previously trained and calibrated with 20 images that did not compose the main sample. For the training sessions, the method of evaluation was explained and reproduced in detail, and the potential difficulties of the methods (e.g. magnification and distortion of the panoramic images) were discussed and taken into consideration. All images were presented to the observers in a blind (without identification) and randomized manner. Image analysis was performed in sets of 30 images per day, in a 24-inch monitor, under dimmed light conditions. All panoramic radiographs were analyzed first and after 30 days the CBCT volumes were assessed in order to avoid the memorization of the cases by the observers. In panoramic radiographs, third molars were visualized with ImageJ software package (National Institute of Health™, Bethesda, MA, USA); the software XoranCAT 3.0 (Xoran Technologies, Ann Arbor, MI, USA) was used to dynamically assess the CBCT scans. The use of enhancement filters as well as the manipulation of bright and contrast were allowed in order to reproduce the clinical practice.

- Variables

**Figure 1:**
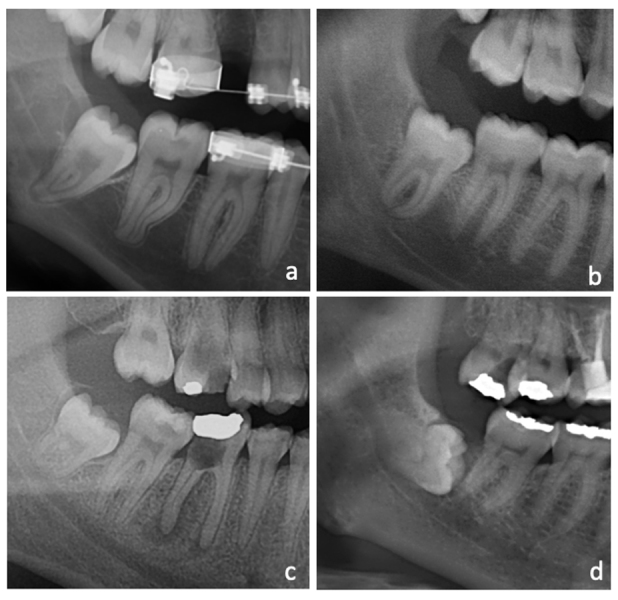
Cutouts of panoramic radiographs showing the absence of marginal bone loss between the second molar and the adjacent third molar (a), slight initial bone loss (b), moderate bone loss reaching the middle third of the second molar root (c) and severe bone loss reaching the apical third of the second molar root (d).

In the first analysis, the third molar position was classified into mesioangular, horizontal, vertical, distoangular, inverted or transverse (11). In the second, the absence or presence of marginal bone loss of the adjacent second molar was recorded. The absence of bone loss was established based on the integrity of the alveolar bone crest, while lack of integrity was used to indicate bone loss. In case of bone loss in panoramic radiographs, it was qualitatively classified according to severity into slight (affecting the coronal third of the second molar root), moderate (reaching the middle third of the second molar root), and severe (reaching the apical third of the second molar root) (Fig. 1).

**Figure 2:**
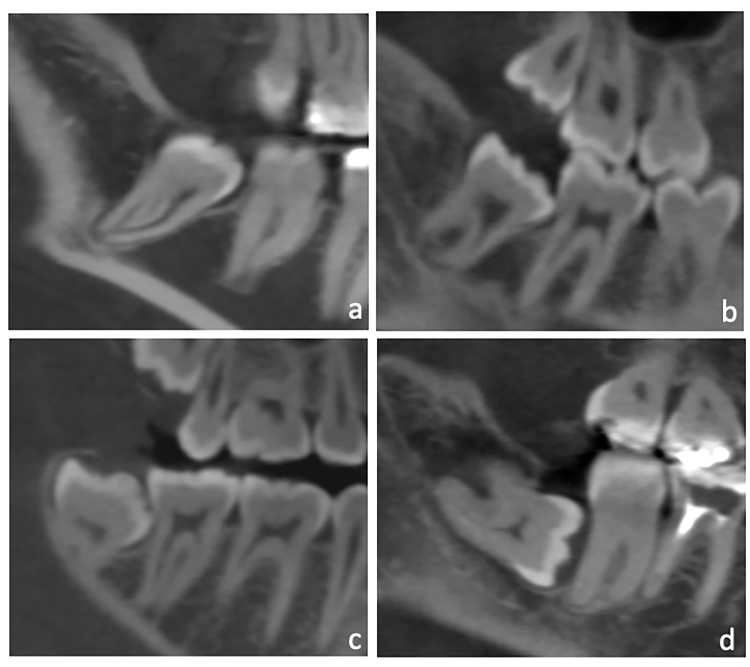
CBCT sagittal slices illustrating the absence of marginal bone loss between the second molar and the adjacent third molar (a), slight initial bone loss (b), moderate bone loss (c) and severe bone loss (d).

In CBCT volumes, the first and second analysis were reproduced, but for the classification of the bone loss severity, the bone defects were quantitatively measured from the cement-enamel junction to the deepest point of the defects. The obtained measurements were converted into categories of bone loss severity, namely: slight (3 – 4 mm), moderate (4 – 6 mm) and severe (>6 mm) (Fig. 2).

- Assessing the risk of bias and statistical analysis

Intra-examiner reproducibility and agreement was assessed by reanalyzing panoramic radiographs and CBCT scans of 40 patients within an interval of 30 days. Agreement between panoramic radiographs and CBCT scans for the presence of bone loss was calculated by means of McNemar test and Kappa statistics. Agreement related to third molar position and classification of bone loss was calculated with Bowker test and Kappa statistics. Kappa values were interpreted as follows: poor agreement <0; very slight agreement 0-0.19, slight agreement 0.2-0.39, moderate agreement 04-0.59, substantial agreement 0.6-0.70, almost perfect agreement 0.8-1. The association between bone loss and sex was tested with Chi-square, while the association between third molar position and bone loss was tested with Fisher’s exact test. Associations of bone loss based on age were investigated with generalized linear models. Examiner agreement was quantified via percentage of agreement and kappa statistics for the classification of presence or absence of bone loss, third molar position, and classification of bone loss severity. Intraclass Correlation Coefficient (ICC) and Bland-Altman test were used to verify the reliability of bone defects measurements in CBCT. Confidence interval was set at 95% and statistical significance at 5%. SAS (SAS Institute™, Cary, NC, USA) and R (R Foundation, Vienna, Austria) software packages were used.

## Results

- Main results

Agreement between image modalities was not detected (*p*<0.05) (Table 1). In panoramic radiographs, bone loss was found in 62.9% of the teeth, while in CBCT scans the prevalence of bone loss reached 80% (Kappa: 0.386). Nearly 29% of the teeth that were classified with absent bone loss in panoramic radiographs were classified with bone loss in CBCT scans. The opposite occurred in 4%.

Agreement on the classification of third molar position was found between panoramic radiographs and CBCT scans (*p*>0.05). Kappa statistics reached 0.783 (Table 2).

Statistically significant differences were found between panoramic radiographs and CBCT scans for the classification of bone loss severity (*p*<0.05) (Table 3). Kappa statistics reached 0.303. The average agreement between image modalities was 41%. In panoramic radiographs 37.1% of the teeth did not reveal bone loss, while 41.9% presented mild, 15.3% presented moderate and 5.6% presented severe bone loss. In CBCT scans, the prevalence of absent, slight, moderate and severe bone loss reached 19.4%, 25.8%, 31.5% and 23.4%, respectively.

**Table 1:**
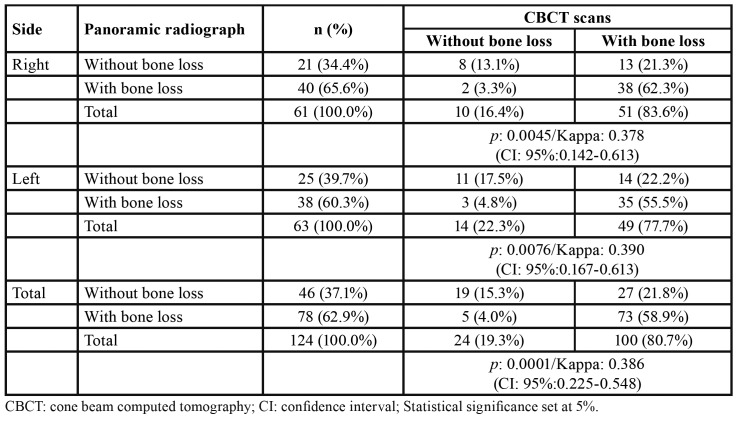
Descriptive analysis of the sample investigated in this study stratified between panoramic radiograph and computed tomography.

**Table 2:**
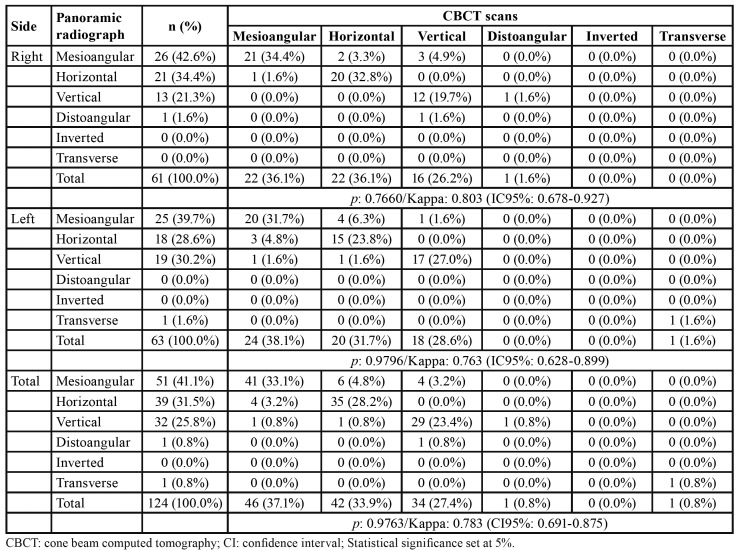
Classification of third molar position in panoramic radiographs and cone beam computed tomography.

**Table 3:**
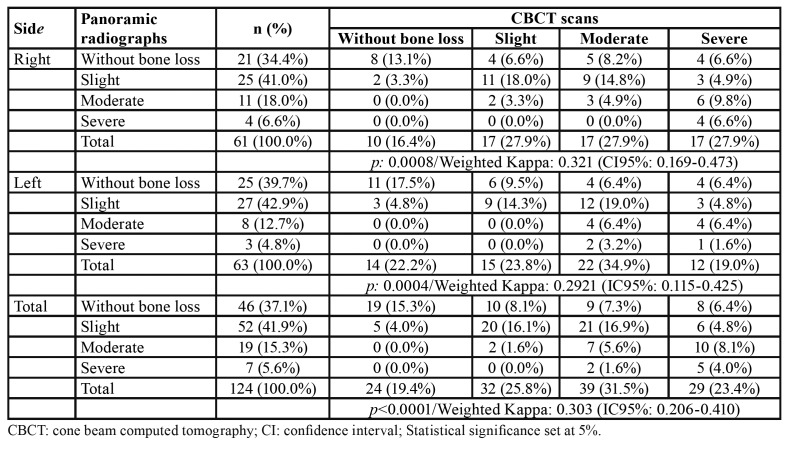
Classification of bone loss severity in panoramic radiographs and cone beam computed tomography.

**Figure 3:**
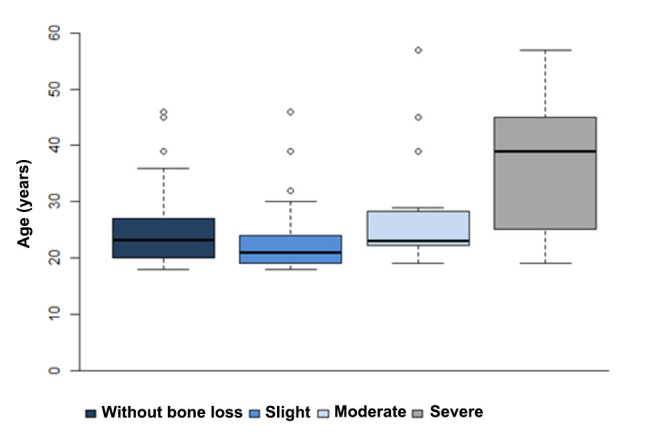
Box plot of age as function of bone loss classification, assessed in CBCT scans.

Sex did not play statistically significant part in bone loss (*p*>0.05). In general, age was not a determining factor for bone loss (*p*>0.05). However, the mean age of patients with severe bone loss was higher than patients with any other bone loss (*p*<0.05) (Fig. 3).

Statistically significant association was detected between third molar position and bone loss (*p*<0.05). In panoramic radiographs, bone loss was found in 80.4% of mesioangular third molars, while 59% and 43.8% was found in horizontal and vertical third molars, respectively. In CBCT scans, the prevalence of bone loss in mesioangular, horizontal and vertical third molars reached 89%, 95.2% and 55.9%, respectively (Table 4).

**Table 4:**
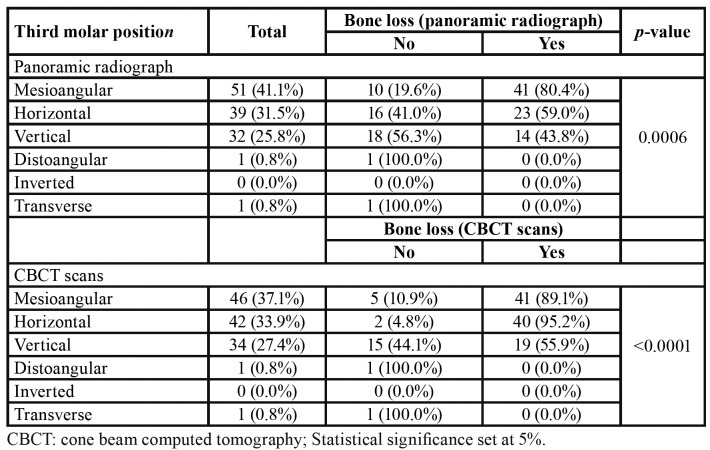
Association between third molar position and bone loss of the adjacent second molar in panoramic radiographs and cone beam computed tomography scans.

- Other analyses

In panoramic radiographs, intra-examiner agreement for the classification of presence/absence of bone loss reached 82.5% (Kappa: 0.527). In CBCT scans, these outcomes reached 92.5% (Kappa: 0.648).

Intra-examiner agreement for the classification of third molar position was 92.5% (Kappa: 0.887) for both panoramic radiographs and CBCT scans.

ICC outcomes showed that the measurements of bone defect in CBCT scans reached excellent intra-examiner agreement of 0.861 with Bland-Altman bias of -0.42.

Intra-examiner agreement for the classification of bone loss severity reached 70% (Kappa: 0.574) in panoramic radiographs and 60% (Kappa: 0.569) in CBCT scans.

## Discussion

Investigating second molar bone loss adjacent to an impact third molar is fundamental to predict tooth prognosis and decide for surgical intervention. In the routine of dentistry, several decisions are taken based on information from panoramic radiographs (1). Despite the evident contributions of these radiographs for the analysis of dental and maxillofacial structures, limitations emerge when detailed information is necessary. This study was designed to compare the assessment of second molar bone loss adjacent to impact third molars between panoramic radiographs and cone beam computed tomography.

When it comes to discussing the methodological set up established in this study, it is important to highlight that ionizing radiation was used with proper clinical justification. In particular, the sample was retrospectively collected from patients that underwent initial evaluation in panoramic radiographs. Because of existing third molar impaction in close relation or superimposed to the mandibular canal, CBCT scans were requested to enable a more predicTable surgical planning. The analysis of panoramic radiographs and CBCT scans was performed and consisted of classifying bone loss in absent or present; measuring bone defects and classifying the bone defect level. The variables were associated with type of exam, third molar angulation, sex and age.

Still regarding the methodological design, some points need to be enlightened, such as the choice for an intra-examiner assessment (and the absence of an inter-examiner assessment), and the quantitative bone loss measurements performed only in CBCT scans. The first is justified by the fact that the observations occurred in simultaneous consensus between two examiners, which resulted in a single measure/decision non-susceptible to inter-examiner assessment. Regarding the quantitative analysis, panoramic radiographs were not measured because of their inherent distortion; unrealistic metric information projected bidimensionally; and overprojection of structures. Moreover, the bone walls of an infrabony defect might jeopardize the determination of its deepest limit. When a reference structure is used, such as second molar’s coronal, middle and apical root parts, metric limitations are tackled and assumptions of bone defect depth become feasible.

The initial outcomes of this study showed a clear difference in the prevalence of bone loss – which reached 62.9% in panoramic radiographs and 80% in CBCT scans. This outcome was expected because CBCT scans enable a three-dimensional and slice-by-slice view of the second and third molars and their relationship with adjacent bone structures, which allow the determination of the bone level in different directions, without the typical superimposition observed in two-dimensional exams of infrabony or combined defects. The recent scientific literature in the field confirms the essential role of CBCT scans as a turning point in clinical decision making process for maxillary third molars management (12). The multiplanar and three-dimensional navigation of CBCT scans Figured as key tools to support clinical decisions.

The comparison between panoramic radiographs and CBCT scans for the analysis of third molars revealed a fair general agreement, however, the former might not perform as accurate as the latter when more detailed analyses are needed, such as to investigate root resorption of the adjacent teeth (13-15). In accordance to previous studies (16), the panoramic radiographs analyzed in the present research reliably enabled the classification of third molar position compared to CBCT scans (Kappa: 0.783). On the other hand, the radiographs were not as accurate to classify bone loss severity. In this context, the agreement between image modalities reached 41%. This outcome has an important impact in the clinical practice because the bone severity classified in panoramic radiographs was underestimated in higher stages of bone loss, namely moderate (15.3%) and severe (5.6%), compared to CBCT. In the latter, the prevalence of moderate (31.5%) and severe (23.4%) bone loss was higher indicating a more detailed view of the second and third molar and their interdental alveolar bone crest. From a clinical point of view, predicting second molar prognosis from the analysis of panoramic radiographs may be challenging and sensitive as it may result in false negatives. In short, undetecTable bone loss may be overlooked.

Bone loss investigated in face of third molar position revealed a higher prevalence among those impacted mesioangular and horizontal. In particular, statistics revealed that 80.4% of the mesioangular third molars were associated with bone loss in panoramic radiographs, while in CBCT scans the prevalence increased to 89%. For horizontal third molars, the prevalence rate of bone loss in panoramic radiographs and CBCT scans reached 59% and 95.2%, respectively. The different prevalence rates of bone loss related to horizontal third molars between panoramic radiographs and CBCT scans (+36.2%) might be explained based on the bidimensional “fitting” of the occlusal surface of the third molars on the distal surface of the second molars – eventually found in panoramic radiographs and potentially hiding bone loss.

These findings follow the current scientific literature that suggests mesioangular and horizontal third molars as those more associated with adjacent lesions (9). More specifically in relation to the prognosis of second molars, both mesioangular and horizontal third molars represent factors to increase the risk of adjacent bone loss (17) and even external root resorption (18). The outcomes of the present study highlight the importance of suspecting of second molar bone loss adjacent to mesioangular angular and horizontally impacted third molars even if clear signs are not visible in panoramic radiographs. Similar suggestions were previously made in the scientific literature for the indication of CBCT scans when suspecting of external root resorption in second molars (14).

Other statistical associations investigated the influence of sex and age on second molar bone loss adjacent to impacted third molars. While sex did not play statistically significant part, age was an important aspect to be considered among patients with severe bone loss; the mean age of patients with severe bone loss was higher than those with slight or moderate bone loss. This finding indicates that patients living with impacted third molars tend to develop with a poor prognosis for second molars. This finding supports the scientific literature that highlights the importance of early radiographic assessment of third molars and eventual extraction under proper indication (15). This scenario may be even worse considering severely angulated third molars. These teeth have not only a bad prognosis for eruption but also represent a risk to adjacent anatomic structures (19,20).

We have shown the reproducibility data by means of percentage of agreement and kappa values given that kappa statistic alone do not express the results obtained from the distribution of the present sample. In other words, the kappa values were not high for most of the evaluated parameters, despite the high percentage of agreement, due to the high prevalence of bone loss in both methods (62.9% panoramic and 80% CBCT).

The present study corroborates the importance of investigating the position of mandibular impacted third molars and their relationship with the adjacent second molars as an important factor in the decision making process involving third molar removal or maintenance. Future studies in the field are encouraged to take a deeper look in the behavior of third molars, especially through retrospective longitudinal analyses, as well as studies in higher levels of evidence evaluating treatment decision and overall impact.

Conclusion

The detection of marginal bone loss on second molars adjacent to impacted third molars and the determination of its severity are more reliable in CBCT scans compared to panoramic radiographs. Furthermore, our results also indicate that second molar bone loss is more prevalent with mesioangular and horizontal third molars, which can support the removal of these teeth, even in the absence of panoramic radiographic signs and clinical symptoms.

## References

[1] Tolentino PHMP, Rodrigues LG, Miranda de Torres É, Franco A, Silva RF (2019). Extractions in patients with periodontal diseases and clinical decision-making process. Acta Stomatol Croat.

[2] Paulander J, Wennström JL, Axelsson P, Lindhe J (2004). Some risk factors for periodontal bone loss in 50-year-old individuals. J Clin Periodontol.

[3] Hermann L, Wenzel A, Schropp L, Matzen L (2019). Marginal bone loss and resorption of second molars related to maxillary third molars in panoramic images compared with CBCT. Dentomaxillofac Radiol.

[4] Reyes-Gilabert E, Luque-Romero LG, Bejarano-Avila G, Garcia-Palma A, Rollon-Mayordomo A, Infante-Cossio P (2017). Assessment of pre and postoperative anxiety in patients undergoing ambulatory oral surgery in primary care. Med Oral Patol Oral Cir Bucal.

[5] Carter K, Worthington S (2015). Predictors of third molar impaction: a systematic review and meta-analysis. J Dent Res.

[6] Garaas R, Moss KL, Fisher EL, Wilson G, Offenbacher S, Beck JD (2011). Prevalence of visible third molars with caries experience or periodontal pathology in middle-aged and older Americans. J Oral Maxillofac Surg.

[7] Nunn ME, Fish MD, Garcia RI, Kaye EK, Figueroa R, Gohel A (2013). Retained asymptomatic third molars and risk for second molar pathology. J Dent Res.

[8] Schmidt JC, Gutekunst CJ, Dagassan-Berndt D, Schmidlin PR, Walter C (2019). Comparison of two-dimensional and three-dimensional radiographs using clinically relevant parameters. Dent J (Basel).

[9] Matzen LH, Schropp L, Spin-Neto R1, Wenzel A (2017). Radiographic signs of pathology determining removal of an impacted mandibular third molar assessed in a panoramic image or CBCT. Dentomaxillofac Radiol.

[10] Von Elm E, Altman DG, Egger M, Pocock SJ, Gøtzsche PC, Vandenbroucke JP (2014). The strengthening the reporting of observational studies in epidemiology (STROBE) statement: guidelines for reporting observational studies. Int J Surg.

[11] Miclotte A, Grommen B, Llano-Pérula MC, Verdonck A, Jacobs R, Willems G (2017). The effect of first and second premolar extractions on third molars: A retrospective longitudinal study. J Dent.

[12] Hermann L, Wenzel A, Schropp L, Matzen LH (2019). Impact of CBCT on treatment decision related to surgical removal of impacted maxillary third molars: does CBCT change the surgical approach?. Dentomaxillofac Radiol.

[13] Hermann L, Wenzel A, Schropp L, Matzen LH (2018). Marginal bone loss and resorption of second molars related to maxillary third molars in panoramic images compared with CBCT. Dentomaxillofac Radiol.

[14] Oenning ACC, Neves FS, Alencar PNB, Prado RF, Groppo FC, Haiter-Neto F (2014). External root resorption of the second molar associated with third molar impaction: comparison of panoramic radiography and cone beam computed tomography. J Oral Maxillofac Surg.

[15] Miclotte A, Franco A, Guerrero ME, Willems G, Jacbos R (2015). The association between orthodontic treatment and third molar position, inferior alveolar nerve involvement, and prediction of wisdom tooth eruption. Surg Radiol Anat.

[16] Brasil DM, Nascimento EHL, Gaêta-Araujo H, Oliveira-Santos C, Almeida SM (2019). Is panoramic imaging equivalent to cone-beam computed tomography for classifying impacted lower third molars?. J Oral Maxillofac Surg.

[17] Matzen LH, Schropp L, Spin-Neto R, Wenzel A (2017). Use of cone beam computed tomography to assess significant imaging findings related to mandibular third molar impaction. Oral Surg Oral Med Oral Pathol Oral Radiol.

[18] Oenning ACC, Melo SLS, Groppo FC, Haiter-Neto F (2015). Mesial inclination of impacted third molars and its propensity to stimulate external root resorption in second molars - a cone-beam computed tomographic evaluation. J Oral Maxillofac Surg.

[19] Vranckx M, Ockerman A, Coucke W, Claerhout E, Grommen B, Miclotte A (2019). Radiographic prediction of mandibular third molar eruption and mandibular canal involvement based on angulation. Orthod Craniofac Res.

[20] De-Azevedo-Vaz SL, Oenning AC, Felizardo MG, Haiter-Neto F, de Freitas DQ (2015). Accuracy of the vertical tube shift method in identifying the relationship between the third molars and the mandibular canal. Clin Oral Investig.

